# Deep learning of noncontrast CT for fast prediction of hemorrhagic transformation of acute ischemic stroke: a multicenter study

**DOI:** 10.1186/s41747-024-00535-0

**Published:** 2025-01-15

**Authors:** Huanhuan Ren, Haojie Song, Shaoguo Cui, Hua Xiong, Bangyuan Long, Yongmei Li

**Affiliations:** 1 Department of Radiology, The First Affiliated Hospital of Chongqing Medical University, Chongqing, China; 2 Department of Radiology, Chongqing University Cancer Hospital, Chongqing, China; 3 College of Computer and Information Science, Chongqing Normal University, Chongqing, China; 4 Department of Radiology, Shapingba Hospital affiliated to Chongqing University (Shapingba District People’s Hospital of Chongqing), Chongqing, China; 5 Department of Radiology, Chongqing General Hospital, Chongqing, China

**Keywords:** Deep learning, Hemorrhage, Ischemic stroke, Thrombolytic therapy, Tomography (x-ray computed)

## Abstract

**Background:**

Hemorrhagic transformation (HT) is a complication of reperfusion therapy following acute ischemic stroke (AIS). We aimed to develop and validate a model for predicting HT and its subtypes with poor prognosis—parenchymal hemorrhage (PH), including PH-1 (hematoma within infarcted tissue, occupying < 30%) and PH-2 (hematoma occupying ≥ 30% of the infarcted tissue)—in AIS patients following intravenous thrombolysis (IVT) based on noncontrast computed tomography (NCCT) and clinical data.

**Methods:**

In this six-center retrospective study, clinical and imaging data from 445 consecutive IVT-treated AIS patients were collected (01/2018–06/2023). The training cohort comprised 344 patients from five centers, and the test cohort included 101 patients from the sixth center. A clinical model was developed using eXtreme Gradient Boosting, an NCCT-based imaging model was created using deep learning, and an ensemble model integrated both models. Comparison with existing clinical scores (MSS, SEDAN, GRASPS) was performed using the DeLong test.

**Results:**

Of the 445 individuals, 202 (45.4%) had HT, 79 (17.8%) had hemorrhagic infarction, and 123 (27.6%) had PH. In the test cohort, the area under the receiver operating characteristic curve (AUROC) of the clinical, imaging, and ensemble model for HT prediction was 0.877, 0.920, and 0.937, respectively. The ensemble model for HT prediction outperformed MSS, SEDAN, and GRASPS scores (*p* ≤ 0.023). The ensemble model predicted PH and PH-2 with AUROC of 0.858 and 0.806, respectively.

**Conclusion:**

Developing and validating an integrated model that can predict HT and its subtypes in AIS patients following IVT based on NCCT and clinical data is feasible.

**Relevance statement:**

The clinical, imaging, and ensemble models based on noncontrast CT and clinical data outperformed existing clinical scores in predicting hemorrhagic transformation of AIS and its subtypes with poor prognosis, facilitating personalized treatment decisions.

**Key Points:**

- The models demonstrated the capability to predict hemorrhagic transformation of acute ischemic stroke quickly, accurately, and reliably.
- The proposed models outperformed existing clinical scores in predicting hemorrhagic transformation.
- The ensemble model provided risk assessment of parenchymal hemorrhage and parenchymal hemorrhage-2 outperforming existing clinical scores.

**Graphical Abstract:**

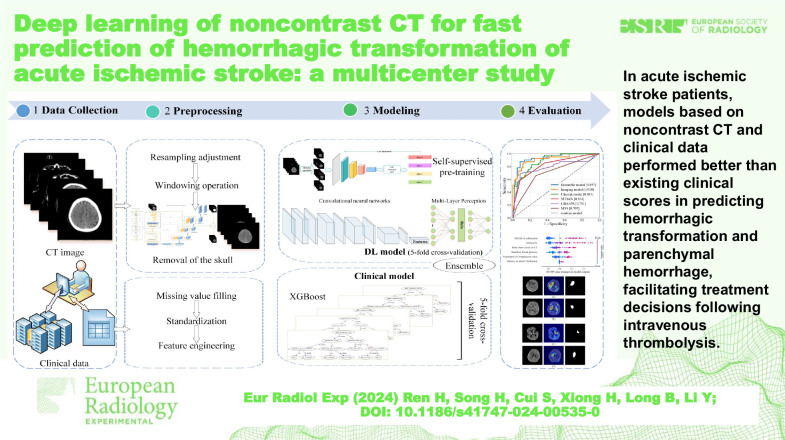

## Background

In acute ischemic stroke (AIS) patients, hemorrhagic transformation (HT) is a significant complication of reperfusion therapy that is associated with poor clinical outcomes [1]. It has been reported that the incidence of HT following intravenous thrombolysis (IVT) ranges from 10 to 48% [2]. The application of IVT is severely limited by this hazardous complication [3]. Accurate risk stratification for HT might help improve the risk-benefit ratio of IVT [4]. Predicting HT is an important issue for stroke practitioners [5].

Previous research has indicated that HT prediction relies on clinical data [6] and various imaging techniques, such as noncontrast computed tomography (NCCT) [7, 8], CT angiography [9], CT perfusion [10], and both conventional and advanced magnetic resonance imaging [11]. Additionally, clinical scores, such as the Multicenter stroke survey (MSS) [12], the sugar, early infarct signs, dense artery, age, and NIHSS (SEDAN) [13], and the glucose at presentation, race, age, sex, systolic blood pressure at presentation, severity of stroke at presentation (GRASPS) [14] have provided much crucial information. Despite these efforts, precise personalized prediction of HT remains challenging [15], complicating clinical decision-making. Therefore, new predictive indicators for HT are needed to optimize treatment strategies.

NCCT offers valuable cerebral information in a shorter time, at a lower cost, with higher patient tolerance, and greater accessibility in emergency situations compared to other methods [16]. Currently, deep learning (DL) is recognized as the most effective machine learning algorithm [17]. Previous studies have utilized DL to identify early cerebral infarction based on NCCT images [18] and predict ischemic stroke outcomes [19]. Although HT prediction has been explored, existing models have not effectively integrated imaging data [20], and research focusing on developing DL models based on NCCT for HT prediction is limited.

According to the European Cooperative Acute Stroke Study—ECASS-I trial, HT is classified into hemorrhagic infarction (HI) and parenchymal hemorrhage (PH), which includes PH-1 (hematoma within infarcted tissue, occupying less than 30%) and PH-2 (hematoma occupying 30% or more of the infarcted tissue) [21]. Notably, the presence of PH, particularly the PH-2 subtype, significantly increases the risk of poor outcomes [22, 23].

Therefore, this study aimed to develop and validate a DL-based model capable of rapidly predicting the risk of HT following IVT using baseline NCCT and clinical data. The focus of our model is on predicting HT as the primary outcome, with PH and its subtypes PH-2 as secondary outcomes.

## Methods

### Patient selection

The institution’s research ethics board approved the retrospective study (CQSC-202309007) in September 2023, and informed consent was waived.

Clinical data and NCCT images from January 2018 to June 2023 were collected. A total of 445 consecutive patients with AIS from six hospitals were included (see Supplementary materials). Among them, 120 patients overlapped with our previous study [24]. Of these, 344 patients (161 with HT, 183 without HT) from five hospitals were divided into the training cohort, while 101 patients (41 with HT, 60 without HT) from the sixth hospital were assigned to the test cohort. The flowchart of patient preparation is depicted in Fig. S1. Of 445 patients, 79 (17.8%) with HI and 123 (27.6%) with PH; further, 38 (8.5%) patients developed PH-1 and 85 (19.1%) developed PH-2; 40 patients (9.0%) developed symptomatic HT.

Patients with AIS were included in this study if they met the following criteria: (1) NCCT examinations performed before IVT therapy; (2) only IVT therapy had been administered following management recommendations for AIS [25–27]; and (3) follow-up NCCT had been conducted within 36 h after IVT therapy. Patients with head trauma injuries, primary cerebral hemorrhage or brain tumors, hemorrhagic infarction at admission, inadequate data, or significant artifacts on NCCT images were excluded.

### Definition and classification of HT

HT was diagnosed based on the following criteria: (1) initial cranial imaging showing no intracranial hemorrhage and subsequent imaging revealing hemorrhage either immediately upon clinical worsening or routinely after 1 week and (2) assessment based on the first imaging examination [21].

HT was classified into HI and PH. HI was further categorized into HI-1, characterized by small petechiae along the infarct margins, and HI-2, marked by confluent petechiae within the infarcted area. PH was categorized into PH-1, where the hematoma occupies less than 30% of the infarcted area, and PH-2, defined by blood clots encompassing 30% or more of the infarcted area [21]. The imaging findings were evaluated by neuroradiologists (R.H.H. and X.H.) blinded to the patients’ details. Any discrepancy was resolved by consensus. The primary outcome of this study was HT and the secondary outcomes were PH and the PH-2 subtype.

### Acquisition and curation of clinical data and NCCT images

Table S1 shows the CT scanners and scanning parameters from the six hospitals; image normalization and skull removal were performed (details in Supplementary materials).

Nineteen clinical features were collected: age, gender, National Institute of Health Stroke Scale (NIHSS) score at admission, history of diabetes mellitus, history of atrial fibrillation, history of stroke, time from onset to CT scan, laboratory examinations (platelets, white blood cells, neutrophils, lymphocytes, monocytes, neutrophil-lymphocyte ratio, lymphocyte-monocyte ratio, eosinophils, hemoglobin, and baseline blood glucose), systolic and diastolic blood pressure. We used MissForest to fill in the missing values in the training and test cohorts, separately (details in Supplementary materials).

### Development of deep learning prediction models

A pretraining model was developed to initialize the DL prediction model’s learnable parameters by transfer learning in the training cohort. In most studies, the ImageNet dataset was used for pretraining [28]. However, this method consists of natural images and can only create the model using the relevant features of natural images rather than medical images. A recent study [29] used research data to conduct self-supervised learning, which effectively improved the performance of the model. In the present study, the same method was employed to pretrain the network (Supplementary materials). This pretraining process enabled the network to learn brain structure features, which was valuable for the following classification tasks. The structure of the pretraining network is shown in Supplementary materials.

The DL predicting model was developed using the training cohort with a DenseNet50-based network architecture, which had four dense blocks. The SwinUNETR, a segmentation network, was initially employed to remove skull interference from the images. To mitigate overfitting due to limited dataset, regularization terms were introduced. Additionally, we leveraged a self-supervised technique involving the prediction of rotation angles during pretraining. Subsequently, we fine-tuned parameters using an Adam optimizer for the classification task. Online data augmentation techniques (TorchIO, https://torchio.readthedocs.io/) served to address the constraints posed by limited data volume (Supplementary materials). Ultimately, a fully connected layer neural network was employed for feature integration following feature extraction, and the probability of HT was generated via the softmax function.

### Development of the clinical model

To avoid overfitting, feature selection was performed in the training cohort. Univariate analysis (T/*U* test) was used to select clinical risk factors for patients with HT. Subsequently, clinical variables with significant difference (*p* < 0.05) were inputted into a multivariate logistic regression to determine independent risk factors for HT. Clinical factors were normalized to 0-to-1 values via the MinMaxScaler. Independent risk factors were used to develop a clinical model via eXtreme Gradient Boosting, as it had previously shown the best performance in predicting HT after IVT using clinical factors [30]. Finally, the clinical prediction model was independently verified in the test cohort.

### Development of the ensemble model

**Fig. 1:**
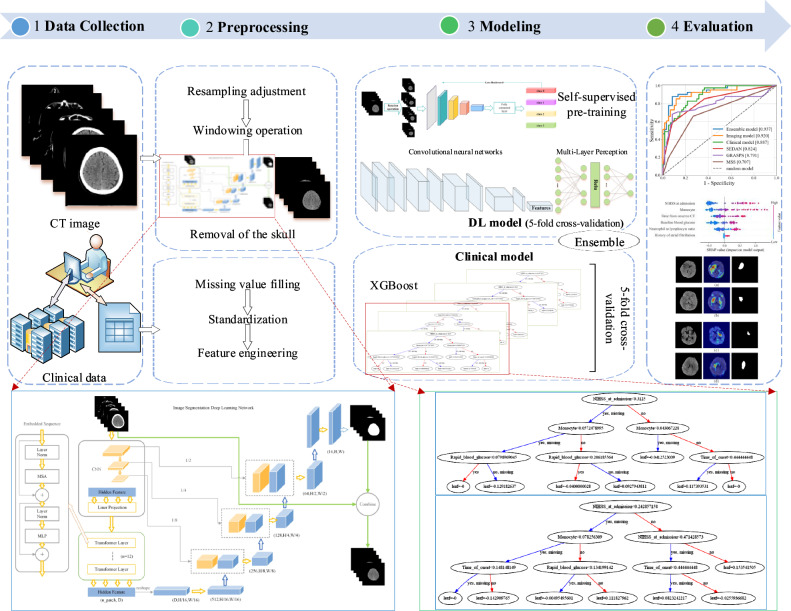
The process of building the machine learning models for hemorrhagic transformation prediction. CT, Computed tomography; DL, Deep learning; XGBoost, Extreme gradient boosting

This study also built an imaging-clinical prediction model (the ensemble model) based on NCCT and clinical data using soft voting. This provided much more types of information and reflected the disease more comprehensively, thus improving predictive performance (Fig. 1).

### Evaluation of the models and statistical analysis

The models’ performance for prediction HT was evaluated in the test cohort using area under the receiving operator curve (AUROC), sensitivity, and specificity. We compared the DL-based models with the existing clinical scores (MSS, SEDAN and GRASPS scores). The Hosmer–Lemeshow test, which compares the consistency between the actual observed outcomes and predicted outcomes of models, was used to evaluate calibration capability.

The performances of the best model for the prediction of PH and PH-2 were evaluated in the test cohort using AUROC, sensitivity, and specificity. Furthermore, we compared the best model with the existing clinical scores (MSS, SEDAN, and GRASPS scores) in the test cohort.

To improve the interpretability of clinical models, the Shapley additive explanation (SHAP) framework was used to calculate the Shapley value for each variable. Regarding deep neural networks, we explored the visualization with Gradient-weighted class activation mapping (Grad-CAM). Grad-CAM explained predictions by unveiling the gradient-weighted contribution of convolutional feature maps in the input space. In practice, the study used the implementation of a slightly improved version of the technique, namely Grad-CAM++.

Statistical analysis was performed using Python software (version 3.7) and R (version 4.1.3). Differences in baseline characteristics of the datasets were assessed with one-way ANOVA or the nonparametric Kruskal–Wallis test. In addition, the Hosmer–Lemeshow test was performed to compare the degree of agreement between predicted and observed outcomes. We considered a two-sided *p* < 0.05 as statistically significant.

## Results

### Demographics

Patient characteristics for the training and test cohorts are detailed in Table 1. Six variables (NIHSS at admission, time from onset to CT scan, monocyte count, baseline blood glucose, neutrophil-lymphocyte ratio (NLR), and history of atrial fibrillation) were identified through univariate and multivariate logistic regression (Supplementary materials).

**Table 1:** Characteristics of patients in the training and test cohorts

Variables	Training cohort (*n* = 344)			Test cohort (*n* = 101)		
	Non-HT (*n* = 183)	HT (*n* = 161)	*p*-value	Non-HT (*n* = 60)	HT (*n* = 41)	*p*-value
Sex, *n* (%)			0.711			1
Male	125 (68.3)	106 (65.8)		39 (65.0)	26 (63.4)	
Female	58 (31.7)	55 (34.2)		21 (35.0)	15 (36.6)	
Age, years, mean ± SD	66.7 ± 12.7	67.1 ± 12.5	0.743	67.1 ± 10.3	69.2 ± 12.9	0.368
History of stroke, *n* (%)			0.002			< 0.001
No	163 (89.1)	122 (75.8)		55 (91.7)	25 (61.0)	
Yes	20 (10.9)	39 (24.2)		5 (8.3)	16 (39.0)	
History of diabetes mellitus, *n* (%)			0.212			0.156
No	134 (73.2)	107 (66.5)		47 (78.3)	26 (63.4)	
Yes	49 (26.8)	54 (33.5)		13 (21.7)	15 (36.6)	
History of atrial fibrillation, *n* (%)			< 0.001			0.002
No	160 (87.4)	111 (68.9)		56 (93.3)	28 (68.3)	
Yes	23 (12.6)	50 (31.1)		4 (6.7)	13 (31.7)	
Time from onset to CT scan, hours, mean ± SD	4.5 ± 2.0	6.1 ± 2.9	< 0.001	4.9 ± 2.4	5.4 ± 2.7	0.309
DBP, mmHg, mean ± SD	87.1 ± 13.7	86.4 ± 14.0	0.606	89.8 ± 13.5	87.2 ± 11.5	0.312
SBP, mmHg, mean ± SD	154.5 ± 25.3	150.2 ± 25.3	0.119	153.3 ± 22.0	154.9 ± 23.7	0.736
Baseline NIHSS, mean ± SD	6.5 ± 5.3	13.3 ± 7.9	< 0.001	4.7 ± 3.7	13.0 ± 6.6	< 0.001
Hgb, g/L, mean ± SD	135.1 ± 21.3	130.5 ± 19.3	0.041	156.9 ± 144.4	132.0 ± 21.7	0.276
PLT, 10^9^/L, mean ± SD	208.3 ± 80.1	206.2 ± 95.9	0.825	210.6 ± 60.1	244.3 ± 81.6	0.019
WBC, 10^9^/L, mean ± SD	7.7 ± 2.4	9.9 ± 3.6	< 0.001	8.0 ± 5.8	9.9 ± 4.3	0.068
Neu, 10^9^/L, mean ± SD	5.5 ± 2.3	7.6 ± 3.5	< 0.001	5.2 ± 1.8	9.1 ± 8.8	0.001
Ly, 10^9^/L, mean ± SD	1.5 ± 0.6	1.5 ± 1.00	0.431	1.5 ± 0.6	1.9 ± 2.9	0.266
Mn, 10^9^/L, mean ± SD	0.5 ± 0.2	0.8 ± 0.9	< 0.001	0.4 ± 0.2	0.7 ± 0.3	< 0.001
NLR, mean ± SD	4.4 ± 3.0	7.4 ± 6.6	< 0.001	4.6 ± 3.5	9.2 ± 14.1	0.017
LMR, mean ± SD	3.6 ± 2.9	2.5 ± 2.4	< 0.001	3.6 ± 1.8	3.3 ± 6.1	0.72
Eon, 10^9^/L, mean ± SD	0.2 ± 0.2	0.1 ± 0.2	0.091	0.1 ± 0.2	0.2 ± 0.3	0.091
Glu, mmol/L, mean ± SD	7.3 ± 3.0	9.0 ± 4.0	< 0.001	7.0 ± 2.7	7.6 ± 2.9	0.289

Categorical variables are presented as numbers (percentage), continuous variables s by mean ± SD

*DBP* Diastolic blood pressure, *Eon* Eosinophils, *Glu* baseline blood glucose, *Hgb* Hemoglobin, *LMR* Lymphocyte-monocyte ratio, *Ly* lymphocytes, *Mn* Monocytes, *Neu* Neutrophils, *NIHSS* National Institute of Health Stroke Scale, *NLR* Neutrophil-lymphocyte ratio, PLT blood platelet, *SBP* Systolic blood pressure, *WBC* White blood cell

### Performance of the models

**Fig. 2:**
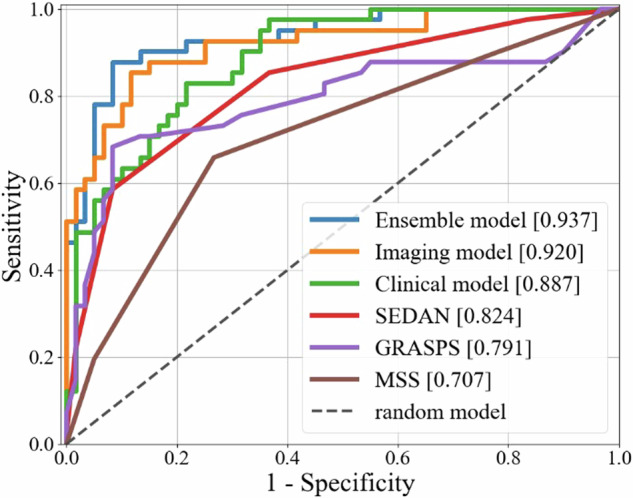
Receiver operating characteristic curves of the three machine learning models and three existing clinical scores (MSS, SEDAN, and GRASPS) for hemorrhagic transformation prediction in the test cohort. The ensemble model is the integration of clinical and imaging models

We integrated five models based on eXtreme Gradient Boosting using the five-fold cross-validation in the training cohort and defined the integrated model as the clinical model. The AUROC, sensitivity, and specificity of the clinical model for HT prediction were 0.887 (95% confidence interval (CI), 0.864–0.910), 0.659, and 0.850, respectively, in the test cohort (Table 2 and Fig. 2).

**Table 2:** Performances of the three deep learning models and three existing clinical scores for hemorrhagic transformation prediction in the test cohort

Models	AUROC	Sensitivity	Specificity
Clinical model	0.887 (0.864–0.910)	0.659	0.850
Imaging model	0.920 (0.897–0.945)	0.854	0.883
Ensemble model	**0.937** (0.908–0.955)	0.878	0.883
MSS	0.707 (0.665–0.739)	0.659	0.733
GRASPS	0.791 (0.747–0.824)	0.585	0.917
SEDAN	0.824 (0.786–0.846)	0.854	0.633

Ensemble model is the integration of clinical and imaging models developed in this study. MSS, SEDAN, and GRASPS scores are three existing clinical scores for predicting hemorrhagic transformation of acute ischemic stroke

*AUROC* Area under the receiver operating characteristic curve

The bold font indicates that the AUC value of the Ensemble model is the highest

The DenseNet models were built based on NCCT images with strategies including regularization, pretraining, and data augmentation (details in Supplementary materials). We integrated the four models based on DenseNet using the five-fold cross-validation in the training cohort and defined the integrated model as the imaging model. The AUROC with a 95% CI, sensitivity, and specificity of the imaging model for HT prediction were 0.920 (95% CI, 0.897–0.945), 0.854, and 0.883, respectively, in the test cohort (Table 2 and Fig. 2).

The integrated model of the clinical and imaging models was defined as the ensemble model. The AUROC with a 95% CI, sensitivity, and specificity of the ensemble model for HT prediction were 0.937 (95% CI, 0.908–0.955), 0.878, and 0.883, respectively, in the test cohort (Table 2 and Fig. 2).

### Comparison of the ensemble model with existing clinical scores for HT prediction

The AUROC of the ensemble model was the highest among the three prediction models in the test cohort. The DeLong test showed that the Ensemble model for HT prediction had superior predictive performance compared to the MSS (*p* < 0.001), the SEDAN (*p* = 0.023), and the GRASPS scores (*p* = 0.008) (Supplementary materials). Calibration capability of the existing clinical scores (MSS, SEDAN, GRASPS) and the ensemble model were assessed.

### Performance of the ensemble model for prediction of PH and PH-2 subtype

**Fig. 3:**
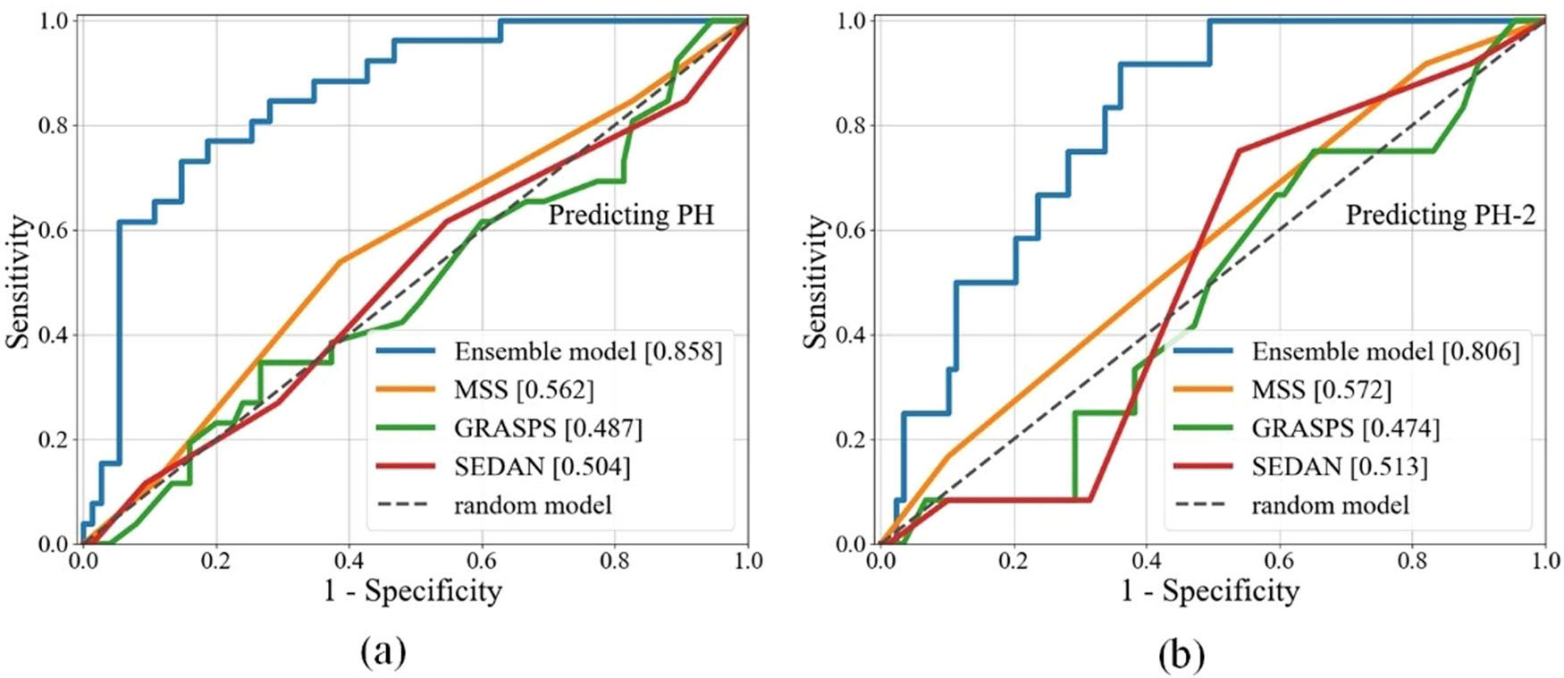
Receiver operating characteristic curves of the three machine learning models and three existing clinical scores for prediction of PH (**a**) and PH-2 (**b**) in the test cohort. Ensemble model is the integration model of clinical and imaging models. MSS, SEDAN, and GRASPS are three of existing clinical scores for predicting hemorrhagic transformation. PH, Parenchymal hemorrhage; PH-2, Hematoma occupying ≥ 30% of the infarcted tissue

The AUROC was 0.858 (95% CI, 0.825–0.893) with 0.769 sensitivity and 0.747 specificity for predicting PH, and 0.806 (95% CI, 0.771–0.839) with 0.750 sensitivity and 0.663 specificity for predicting PH-2 using the ensemble model in the test cohort (Table 3 and Fig. 3).

**Table 3:** Prediction of PH and PH-2 using the ensemble model in the test cohort

	AUROC	Sensitivity	Specificity
PH	0.858 (0.825–0.893)	0.769	0.747
PH-2	0.806 (0.771–0.839)	0.750	0.663

*AUROC* Area under the receiver operating characteristic curve, *PH* Parenchymal hemorrhage, *PH-2* hematoma occupying ≥ 30% of the infarcted tissue

### Interpretation and visualization of clinical and imaging models

**Fig. 4:**
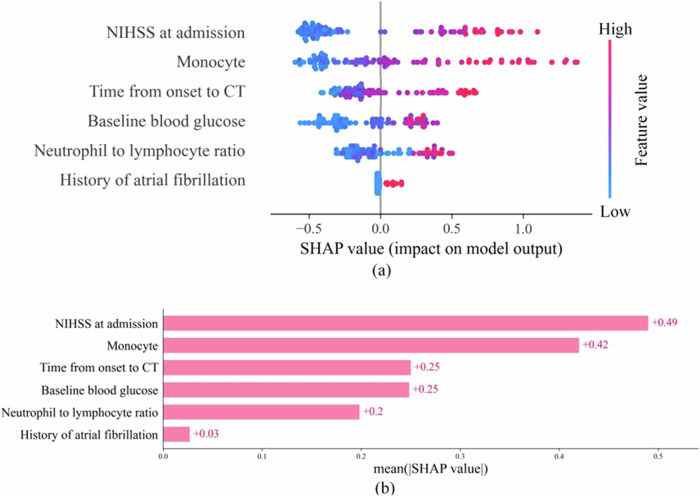
SHAP plots of the clinical model for hemorrhagic transformation prediction. The plots illustrate feature relevance and attribution to the model’s predictive performance. **a** The vertical axis indicates the features, ordered top down, from the most to the least important predictors. The horizontal axis indicates the SHAP values. Each point on the plot represents a Shapley value for a feature. Pink color indicates a positive association with hemorrhagic transformation; blue color indicates a negative association with hemorrhagic transformation. Data points clustered at the vertical line where the Shapley value axis is zero suggest the predictors have little influence on the prediction. **b** The standard bar graph was created by averaging the absolute shape values of each feature

Figure 4a illustrates the SHAP summary plot, highlighting feature importance for all 101 participants in the test cohort. Each dot represents feature attribution in calculating the output risk for each observation. Figure 4b displays the average absolute SHAP value as a bar chart, arranging features by clinical features, and emphasizing their contributions to the model. The SHAP plots illustrate that all six factors (NIHSS at admission, monocyte count, baseline blood glucose, NLR, history of atrial fibrillation, and the time from onset to CT) were positive predictors of HT. The NIHSS at admission was the most important feature for predicting HT.

**Fig. 5:**
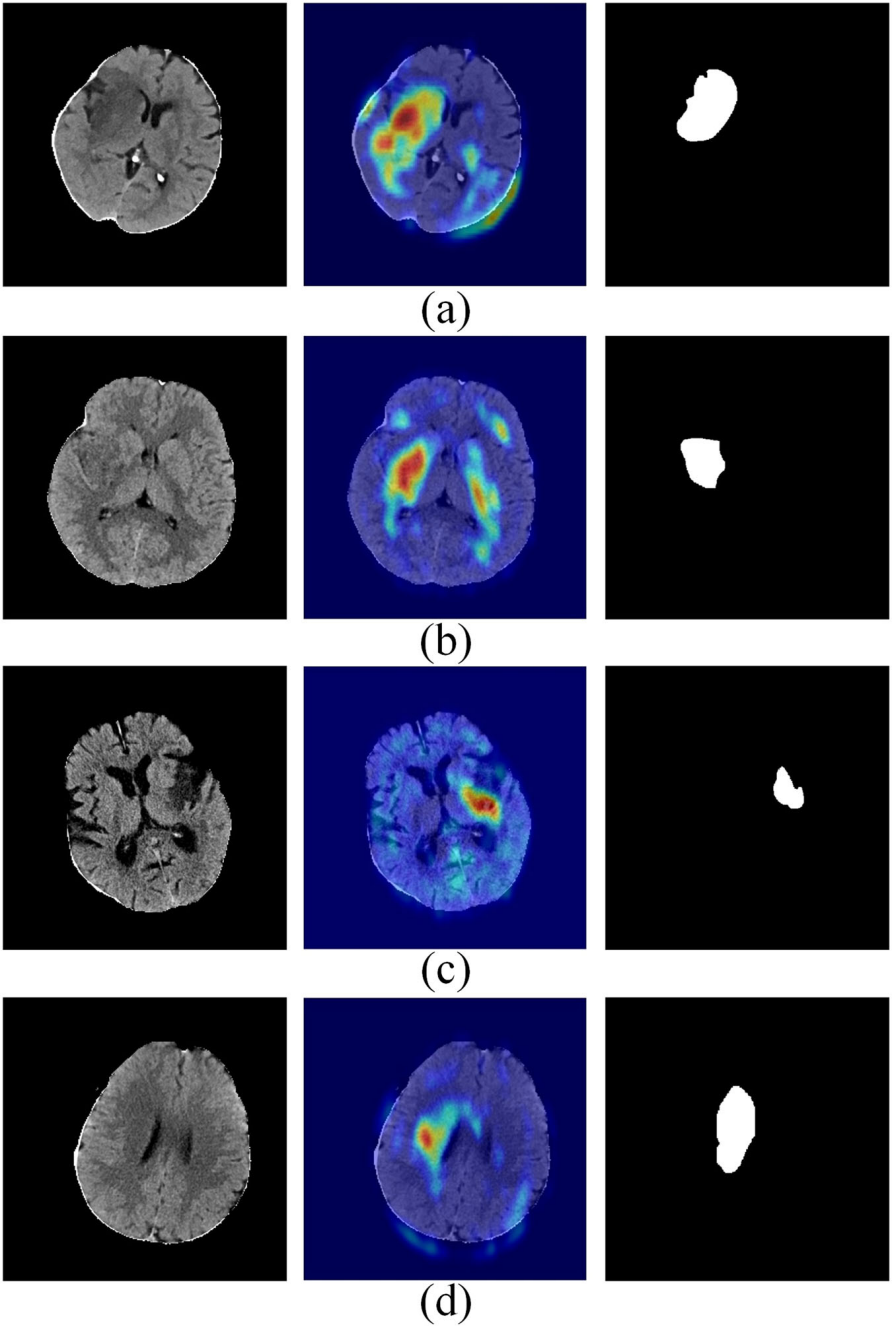
Visualization of the imaging model for hemorrhagic transformation prediction using the Grad-CAM++ method. A 74-year-old man (6 h from onset to CT scan) (**a**) and A 66-year-old woman (3.5 h from onset to CT scan) (**b**) were confirmed to have undergone hemorrhagic transformation based on follow-up images. A 78-year-old man (9 h from onset to CT scan) (**c**) and a 58-year-old woman (3 h from onset to CT scan) (**d**) were confirmed to have had a stroke without hemorrhagic transformation. The first column shows the original image, the second shows the mask of the lesion, and the third shows the class activation map. Darker red indicates a greater contribution for hemorrhagic transformation prediction, while darker blue indicates a lower contribution in the class activation map

In addition, we applied the Grad-CAM++ technique to DenseNet to overcome the lack of diaphaneity. The algorithm visualized feature extraction as heatmaps. Figure 5 shows the heatmaps indicating that the network focused on the infarction and its surrounding areas.

## Discussion

We developed and validated an ensemble model based on clinical data from admission and NCCT images to predict the risk of HT after IVT for patients with AIS, which outperformed the existing clinical scores. Furthermore, the ensemble model also successfully identified patients at a high risk for PH and PH-2 subtype.

Previous studies have concentrated on the risk stratification of HT post-AIS. Some studies have exclusively utilized clinical data, while others, even when incorporating imaging data, restricted their focus to the size of cerebral infarction, overlooking details of CT performed before HT [15]. Predictive models based on structural or functional magnetic resonance imaging data have been developed [30, 31], but the wider application of magnetic resonance imaging is restricted because of its contraindications, longer duration, and general difficulties in performance in the AIS setting. Research has often been confined to patients undergoing intraarterial thrombectomy, leaving a gap in understanding the HT risk in patients receiving IVT [30]. Furthermore, symptomatic HT had been utilized as a predictive outcome in certain studies [30]; nevertheless, literature findings indicated that the prognosis of patients may also be impacted by the asymptomatic HT [5]. Therefore, this study aimed to stratify HT risk in IVT patients, with secondary objectives of predicting PH and the PH-2 subtype, both associated with poor prognoses.

In our study, the selected clinical factors were determined via pathophysiological insights, clinical experience, and literature review, and the time required to obtain the data. After univariable and multi-variable analysis, six key features were chosen: NIHSS at admission, time from onset to CT scan, monocyte count, baseline blood glucose, NLR, and history of atrial fibrillation. NIHSS at admission emerged as the most significant predictor. Previous studies corroborate our findings, highlighting NIHSS score and atrial fibrillation history as HT-independent predictors [32]. Hiong’s analysis [33] found that elevated blood glucose can accelerate blood-brain barrier disruption, leading to the occurrence of HT. Monocytes can produce and release proinflammatory cytokines such as interleukin-6 and IL-1β [34], which increase the risk of HT. NLR reflects the balance between neutrophil and lymphocyte levels. NLR has also been shown to have a positive association with an increased risk of HT, which is consistent with our research [35]. Studies have reported that the onset time of stroke does not predict HT [36] where our findings suggest this is an important variable. However, this result may have been influenced by participant differences, and further investigation is therefore warranted. Based on these six factors, the AUROC of the clinical model we established was 0.887, indicating that these factors can provide accurate predictive information for HT from the patient’s overall state.

The DL-based imaging model extended beyond lesion analysis, incorporating multidimensional data from the stroke area and its surroundings, including size, density, and heterogeneity. It also took into account the fact that certain background appearances on brain CT scans were associated with certain known predictors (such as small-vessel ischemia, leukoaraiosis, and atrophy) of HT [37, 38]. While Ru’s study applied DL to predict HT risk in patients with onset times under 6 h [37], our model broadened its applicability to AIS patients experiencing wake-up strokes and those with indeterminate onset times.

Additionally, the DL prediction model established in our study was end-to-end. Although the DL features were latent, we have taken steps to enhance the model’s interpretability. First, model architecture transparency: While deep neural networks are inherently “black box” models, revealing the structure and function of each network layer can shed light on the inner workings of the model. Second, visualization techniques: image visualization techniques such as Grad-CAM and “occlusion sensitivity” can be employed to interpret the decision-making process of the deep learning model in image classification tasks. Therefore, in this study, we aimed to help readers better understand the underlying mechanisms of the model by displaying the model’s structure and parameters. We also performed a Grad-CAM interpretability analysis. The heatmap in the second column of Fig. 5 demonstrates that the regions of interest identified by the DL prediction model partially overlap with the radiologist’s manually delineated lesion areas (third column), which are critical regions that influence adverse complications or patient prognosis.

The ensemble model was developed to integrate data from whole-body and brain tissue, offering a comprehensive assessment of HT risk. Calibration curve analysis confirmed a strong concordance between the Ensemble model’s predictions and actual outcomes (*p* = 0.061, Hosmer–Lemeshow test), demonstrating its validity. The ensemble model can predict PH and its subtype PH-2, and both outperformed the existing clinical scores.

Despite the promising results, the study had some limitations: (1) a larger sample size is required for DL even though data augmentation was performed; (2) the model established in this study did not differentiate between symptomatic or asymptomatic HT, nor did it predict clinical outcomes; (3) theoretically, we should understand the full reasoning behind the results; however, although the models selected the most important clinical features and concentrated on the lesion and perilesional areas of the NCCT images, it is still not fully clear how this was achieved, being this an end-to-end DL model offering several advantages, including reducing cumulative errors, simplifying the workflow, and minimizing reliance on specialist expertise; (4) more medical institutions are required to validate the generality of our model before it can be extensively used in clinical settings, as this study only used data from one medical institution as the test set.

In conclusion, we developed an integrated model that predicted the risk of HT and its subtypes with bad prognosis (PH and PH-2) in AIS patients following IVT based on NCCT images and commonly used clinical data. The rapid prediction demonstrated the potential to assist clinicians in making treatment decisions that outperformed existing clinical scores.

## Supplementary information

**Additional file 1: Figure S1**. The flowchart of patients’ preparation. **Figure S2**. The process of skull removal. **Figure S3**. The process of pretraining. **Figure S4**. Changes in three parametres during pretraining. **Figure S5**. The correlation matrix for the six clinical characteristics. **Table S1**. Models of CT Scanners and Scanning Parameters for Six Institutions. **Table S2**. Performances of the XGB models based on different clinical features in the test cohort. **Table S3**. Performances of DenseNet models developed using different strategies in the test cohort. **Table S4**. The results of DeLong test of the three machine learning models for HT prediction and the existing clinical scores (MSS, SEDAN and GRASPS) in the test cohort.

## Data Availability

The datasets generated and/or analyzed during the current study are not publicly available because the subjects did not provide written consent for their data to be publicly shared. However, datasets can be obtained by request from specific research groups.

## Abbreviations

AIS: Acute ischemic stroke
AUROC: Area under the receiver operating characteristic curve
CI: Confidence interval
CT: Computed tomography
DL: Deep learning
Grad-CAM: Gradient-weighted class activation mapping
GRASPS: Glucose at presentation, race, age, sex, systolic blood pressure at presentation, severity of stroke at presentation
HI: Hemorrhagic infarction
HT: Hemorrhagic transformation
IVT: Intravenous thrombolysis
MSS: Multicenter stroke survey
NCCT: Noncontrast CT
NIHSS: National Institute of Health Stroke Scale
NLR: Neutrophil-lymphocyte ratio
PH: Parenchymal hemorrhage
SEDAN: Sugar, early infarct signs, dense artery, age, and NIHSS
SHAP: Shapley additive explanation
