# Supplementary material for: Deep learning of noncontrast CT for fast prediction of hemorrhagic transformation of acute ischemic stroke: a multicenter study
Source: Eur Radiol Exp. 2025 Jan 15;9:8. doi: 10.1186/s41747-024-00535-0 (PMC11735721; doi:10.1186/s41747-024-00535-0)
Supplement: Supplementary file 1 — Additional file 1: Figure S1. The flowchart of patients’ preparation. Figure S2. The process of skull removal. Figure S3. The process of pretraining. Figure S4. Changes in three parametres during pretraining. Figure S5. The correlation matrix for the six clinical characteristics. Table S1. Models of CT Scanners and Scanning Parameters for Six Institutions. Table S2. Performances of the XGB models based on different clinical features in the test cohort. Table S3. Performances of DenseNet models developed using different strategies in the test cohort. Table S4. The results of DeLong test of the three machine learning models for HT prediction and the existing clinical scores (MSS, SEDAN and GRASPS) in the test cohort. [file 41747_2024_535_MOESM1_ESM.pdf]

Deep learning of noncontrast CT for fast prediction of hemorrhagic transformation of acute ischemic stroke: a multicenter study

ELECTRONIC SUPPLEMENTARY MATERIAL

Supplementary material 1

Data were from the following six hospitals: 1) the First Affiliated Hospital of Chongqing Medical University; 2) Chongqing General Hospital; 3) Haikou Affiliated Hospital of Central South University Xiangya School of Medicine; 4) the Second People's Hospital of Hunan Province/Brain Hospital of Hunan Province; 5) Changsha Central Hospital (the Affiliated Changsha Central Hospital, Hengyang Medical School, University of South China); 6) People's Hospital of Yubei District.

Supplementary material 2

The flowchart of patients' preparation was depicted in Figure S1.

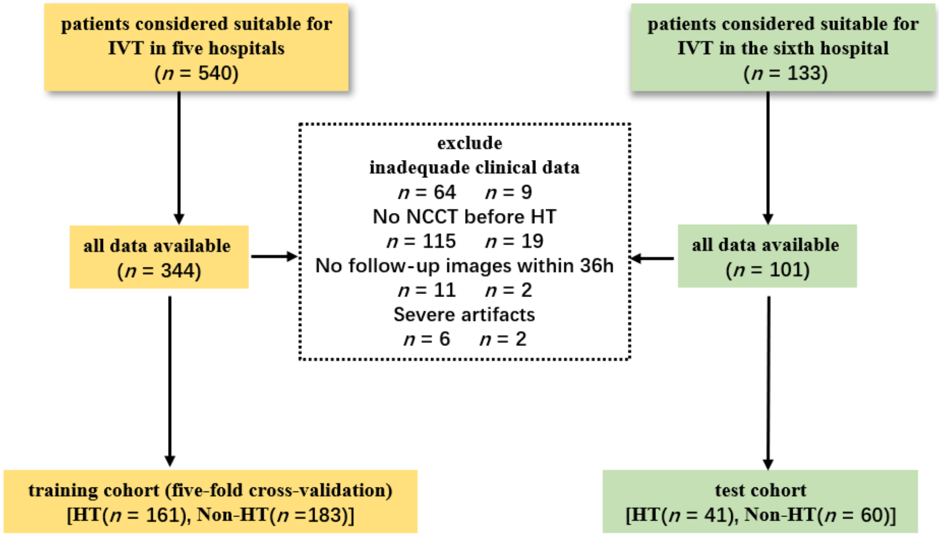

Figure S1 The flowchart of patients' preparation

Note: IVT, intravenous thrombolysis; HT, hemorrhagic transformation.

Supplementary material 3

Table S1 Models of CT Scanners and Scanning Parameters for Six Institutions

Center	equipment	Tube voltage (KV)	Tube current (mA)	Matrix size	Slice thickness (mm)	Slice Spacing (mm)	In-plane space resolution (10% MTF) (lp/cm)
1	Canon Aquilion one	130	90-170	512×512	5	5	22.9#
2	Siemens SOMATOM definition Flash	120	150-190	512×512	5	5	≧13.5
3	UNITED IMAGING uCT 760	120	160-230	512×512	5	5	≧10
4	Philips Brilliance iCT	120	200-240	512×512	5	5	13
5	GE LightSpeed VCT	120	230-300	512×512	5	5	8.5
6	Siemens SOMATOM definition Flash	120	180-235	512×512	5	5	≧13.5

Notes: # indicates that since the white paper for this device does not provide the MTF at 10%, we have instead presented the MTF at 2%. *MTF Modulation Transfer Function.* The MTF at 10% corresponds to the values obtained using the standard algorithm.

Supplementary material 4

Data Preprocessing

Method of filling in missing values

Clinical data generally had minimal missing rates. Only 0.29% of the baseline blood glucose measurements in the training cohort were lacking, and 0.99% of the diabetes history and the time of onset values in the test cohort were missing.

To avoid data leakage, the MissForest algorithm[1] was applied after the data had been divided, filling in the missing values in the training set and the test set, respectively. This approach was chosen because the MissForest algorithm produces excellent results when both continuous and categorized variables are filled in at once. Only classification variables can be dealt with by K-Nearest Neighbor. Even the multiple imputation requires restrictions on the distribution of data and the choice of parameter models, whereas MissForest does not and has a broader range of applications.

The Preprocessing of NCCT Images

Image normalization

All images were first resampled to isotropic voxels of 1 mm^3 using B-spline interpolation. Then we converted all DICOM format into NII format using SimpleITK (https://simpleitk.org), and Hounsfield units were windowed using the brain window (window level = 40 HU, window width = 80 HU). We linearly compressed the pixel intensity range into [0, 255], and normalized it to 0 to 1 using the MinMaxScaler technique. Then the skull was removed from all the images. Again, we compressed the pixel intensity range into [0, 255], and normalized it to 0 to 1 using the MinMaxScaler technique.

The process of skull removal

We trained 100 epochs on the Swin UNETR[2] model using 125 segmented data from the Neurofeedback Skull-striped dataset (NFBS_Dataset) (http://preprocessed-connectomes-project.org/NFB_skullstripped/) until its results converged. The network parameters could then be adjusted after employing the CT data from the 24 patients in this study for transfer learning. The segmentation process and results were shown in Figure S2. After that, the interference of the skull was removed.

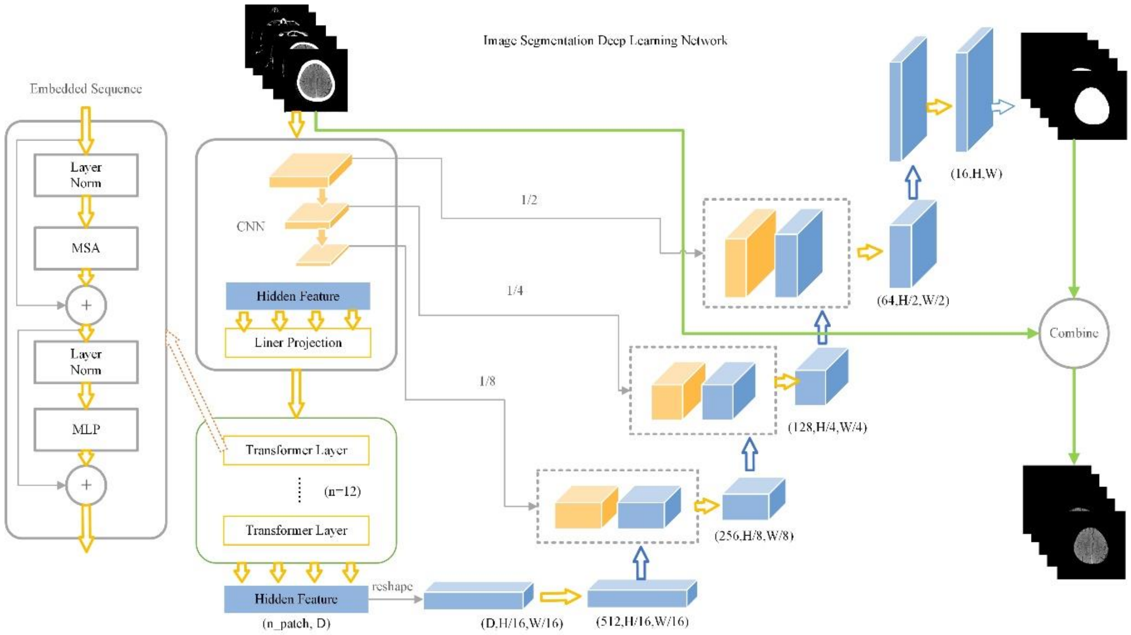

Figure S2 The process of skull removal

Supplementary material 5

The process of pre-training

During pre-training, we rotated the input of our images clockwise in space in one of four ways (0◦, 90◦, 180◦, 270◦), and the images were labeled with the number of 90° of image rotations (0, 1, 2, 3). Then the generated image data was divided into a training cohort and a test cohort at the ratio of 8:2 to train the pre-training model. In the pre-training process (Figure S3), the initial weight of the model was set by common methods[3]. We set the batch size as 2 and the total number of iterations as 200. According to the training and validation loss curves (Figure S4), the model converged at the 74th epoch. Later, the weight parameters of the 74th epoch were used to initialize the 3D convolutional layers of the deep learning classification model. However, the common initialization method, in which the weight was set to 1 and the bias to 0, was still used by the final fully connected neural networks (FCNN) classifier.

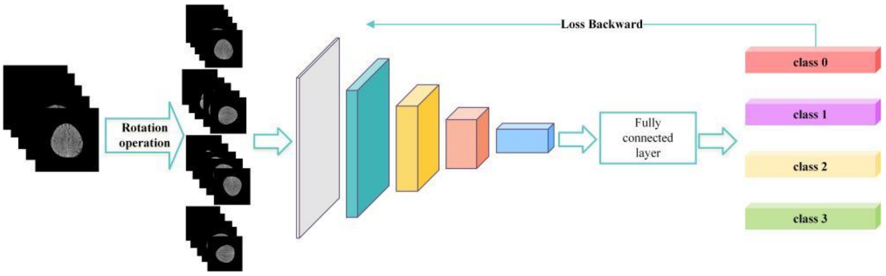

Figure S3 The process of pre-training

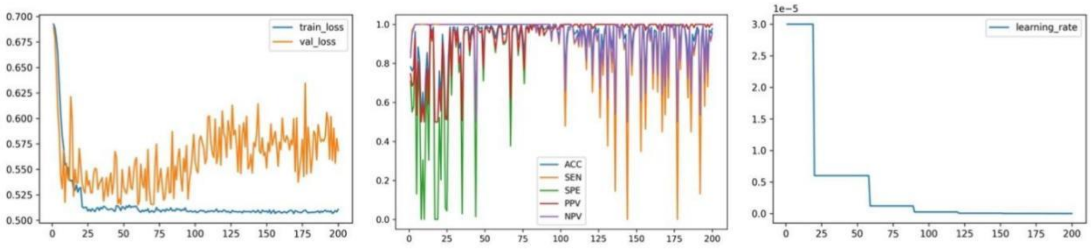

Figure S4 Changes in three parametres during pre-training

Note: The loss function is shown on the left, the pre-training model's evaluation index on the middle, the learning rate on the right.

Supplementary material 6

Regularization & Mixed Loss Strategy of deep learning models

To assess the difference between the probability distribution obtained by the current training and the true distribution, cross-entropy was used. It described the distance between the actual output (probability *p*) and the expected output (probability *q*), that was, the smaller the value of cross-entropy, the closer the two probability distributions were. For the formula, see (1).

$$Loss\_CE = -\sum_{i=1}^{n} q(x_i)\, log(p(x_i)) \quad (1)$$

We used $L1$ and $L2$ regularization. The weight attenuation of parameter norm penalty was used in the optimizer. In addition, the final loss function was built by adding regularization items (2) and (3) to the target function:

$$L2\_loss = \frac{1}{2}\|w\|_2^2 \quad (2)$$

$$L1\_loss = \|w\|_1 = \sum_i |w_i| \quad (3)$$

$$Loss = Loss\_CE + \lambda_1 L1\_loss + \lambda_2 L2\_loss \quad (4)$$

w is the model parameter, λ_1 and λ_2 are the factors that control the degree of punishment. The factor size is a super parameter that was manually adjustable. The feature weight with low covariance with the output target would shrink when using $L2$ regularization, however, $L1$ regularization would result in more sparse solutions than $L2$ and certain parameters will be zero. As a result, introducing regularization would efficiently solve the over-fitting issue with DL models on short data sets and efficiently enhance the model's generalizability. Additionally, even though the number of positive and negative samples in the data set was near, there were still a few differences. To address these few differences, the weight parameter was employed in the cross-entropy loss function.[4]

Supplementary material 7

Data Augmentation

Methods of data augmentation included Random Flip, Random Affine, Random Elastic Deformation, and so on. The probability of occurrence of spatial transforms and intensity transforms is 0.2.

Supplementary material 8

the Optimizer of Deep Learning Models

An Adam optimizer[5] was used to find optimal parameters for the Imaging Model, the learning rate was set to 3×10^{-5} at the beginning, and weight attenuation was 0.1. The learning rate dynamic adjustment strategy was the ReduceLROnPlateau method in Python. The following 20 epochs would not monitor the verification loss if the verification loss did not drop for 10 epochs, at which point the learning rate will be multiplied by 0.2 to generate the new learning rate. Here, the drop-off level was 1×10^{-4}. The minimum learning rate was set to 1×10^{-12}. NVIDIA GeForce RTX 3080 Ti graphics cards with 24 GB of memory were used in the development process. We used the cross-entropy loss function to develop the network. The DL model was finally developed with 180 epochs. Pytorch (1.10.2) and Python (3.7.11) were the main tools used in our experiments.

Supplementary material 9

Performances of the XGB models based on different clinical features

The XGB model was named as XGB1 based on all the 19 clinical features, XGB2 based on the 13 features obtained through t-test or U test screening, and XGB3 based on the six variables [NIHSS at admission, time from onset to CT scan, number of monocytes, baseline blood glucose, neutrophil-lymphocyte ratio (NLR) and history of atrial fibrillation]

In identifying patients with HT, the XGB1 showed an AUC of 0.867, an AUC of 0.856 for XGB2 and an AUC of 0.877 for XGB3 (Table S2).

Table S2 Performances of the XGB models based on different clinical features in the test cohort

Model	AUC	Sensitivity	Specificity
**XGB1**	0.867	0.727	0.803
**XGB2**	0.856	0.702	0.823
**XGB3**	**0.877**	0.717	0.837

Note: XGB, eXtreme Gradient Boosting; AUC, the area under the ROC curve.

Performances of DenseNet prediction models developed using different strategies

Each deep learning model in Table S3 was validated in the test cohort after undergoing a five-fold cross-validation in the training cohort.

Table S3 displays that DenseNet4 with data enhancement and pre-training (AUC = 0.886, sensitivity = 0.781, specificity = 0.840) performed better than the other four DenseNet models in the test cohort.

Table S3 Performances of DenseNet models developed using different strategies in the test cohort

Model	AUC	Sensitivity	Specificity
**DenseNet1**	0.842	0.702	0.803
**DenseNet2**	0.868	0.761	0.787
**DenseNet3**	0.875	0.698	0.857
**DenseNet4**	**0.886**	0.781	0.840

Note: **DenseNet1** is a classification model based on NCCT images after skull removal; **DenseNet2** is a classification model after adding regularization based on DenseNet1; **DenseNet3** is a classification model based on DenseNet2 after pre-training; **DenseNet4** is a classification model based on DenseNet3 after data enhancement. AUC, the area under the ROC curve.

Supplementary material 10

Model evaluation

The DeLong test showed that the Ensemble model had better performance for HT prediction than the MSS score (p < 0.0001), the SEDAN score (p = 0.023) and the GRASPS score (p = 0.0078) (Table S4).

Table S4 The results of DeLong test of the three machine learning models for HT prediction and the existing clinical scores (MSS, SEDAN and GRASPS) in the test cohort

models	**Ensemble model**	Clinical model	Imaging model	SEDAN	GRASPS	MSS
Ensemble model	1	0.0898	0.0965	0.023	0.0078	< 0.0001
Clinical model	0.0898	1	0.3562	0.1797	0.0496	0.0003
Imaging model	0.0965	0.3562	1	0.0613	0.0257	0.0002
SEDAN	**0.023**	0.1797	**0.0613**	1	0.4815	0.0089
GRASPS	**0.0078**	**0.0496**	**0.0257**	0.4815	1	0.019
MSS	< **0.0001**	**0.0003**	**0.0002**	0.0089	0.019	1

We assessed the calibration ability of the DL-based prediction models by comparing the consistency between the actual observed outcomes and predicted outcomes. For all the three models (clinical, imaging and ensemble models), the fit between the predicted values and the observed values were analyzed in the test cohort (p = 0.4471, p = 0.0664, p = 0.0612, respectively; Hosmer-Lemeshow test).

Supplementary material 11

The clinical characteristics following screening do not manifest collinearity, as shown in Figure S5.

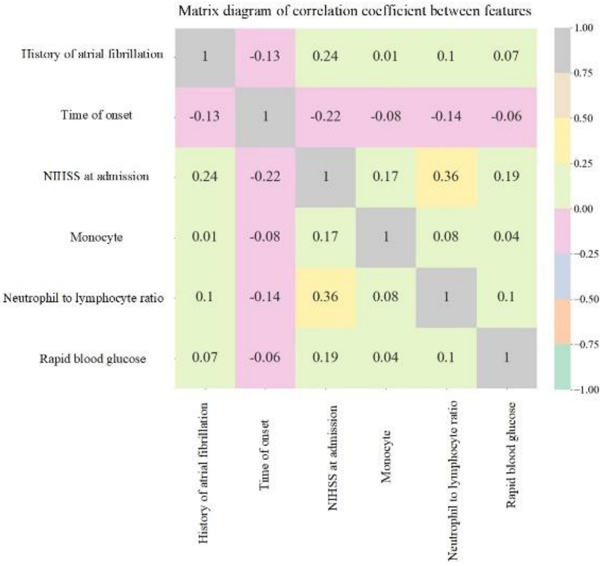

Figure S5 The correlation matrix for the six clinical characteristics

Note: The names of the six clinical features are displayed on the left and below, the correlation values are represented by the numbers in the tiny square, and the color bar is displayed on the right.

1. Stekhoven DJ, Bühlmann P. MissForest--non-parametric missing value imputation for mixed-type data. *Bioinformatics* 2012;28(1):112-118.
2. Hatamizadeh A, Nath V, Tang Y, et al. Swin UNETR: Swin Transformers for Semantic Segmentation of Brain Tumors in MRI Images. In: Cham, 2022, pp.272-284. *Springer International Publishing*.
3. He K, Zhang X, Ren S, et al. Delving Deep into Rectifiers: Surpassing Human-Level Performance on ImageNet Classification. In: *2015 IEEE International Conference on Computer Vision* (ICCV) 7-13 Dec. 2015 2015, pp.1026-1034.
4. Xu L, Yang C, Zhang F, et al. Deep Learning Using CT Images to Grade Clear Cell Renal Cell Carcinoma: Development and Validation of a Prediction Model. *Cancers (Basel)* 2022;14(11):2574.
5. Kingma DP, Ba J. Adam: A Method for Stochastic Optimization. *CoRR* 2014;abs/1412.6980.
